# Liver Proteome Alterations in Red Deer (*Cervus elaphus*) Infected by the Giant Liver Fluke *Fascioloides magna*

**DOI:** 10.3390/pathogens11121503

**Published:** 2022-12-08

**Authors:** Karol Šimonji, Dean Konjević, Miljenko Bujanić, Ivana Rubić, Vladimir Farkaš, Anđelo Beletić, Lea Grbavac, Josipa Kuleš

**Affiliations:** 1 Internal Diseases Clinic, Faculty of Veterinary Medicine, University of Zagreb, 10 000 Zagreb, Croatia; 2 Department of Veterinary Economics and Epidemiology, Faculty of Veterinary Medicine, University of Zagreb, 10 000 Zagreb, Croatia; 3 Laboratory of Proteomics, Internal Diseases Clinic, Faculty of Veterinary Medicine, University of Zagreb, 10 000 Zagreb, Croatia; 4 Department of Parasitology and Parasitic Diseases with Clinic, Faculty of Veterinary Medicine, University of Zagreb, 10 000 Zagreb, Croatia; 5 Department of Chemistry and Biochemistry, Faculty of Veterinary Medicine, University of Zagreb, 10 000 Zagreb, Croatia

**Keywords:** wildlife, liver fluke, host-pathogen interaction, proteomics, tandem mass tag

## Abstract

Liver fluke infections are recognised as diseases with worldwide distribution and considerable veterinary and public health importance. The giant liver fluke, *Fascioloides magna*, is an important non-native parasite which has been introduced to Europe, posing a threat to the survival of local wildlife populations such as red deer (*Cervus elaphus*). The aim of the study was to analyse differences in liver proteomes between *F. magna*-infected and control red deer groups using a label-based high-throughput quantitative proteomics approach. The proteomics analysis identified 234 proteins with differential abundance between the control and infected groups. Our findings showed that *F. magna* infection in this definitive host is associated with changes in the metabolism of proteins and fatty acids, oxidative stress, fibrosis, and signaling pathways. The identified proteins and associated biological pathways represent a valuable contribution to the understanding of host–parasite interactions and the pathogenesis of liver fluke infection.

## 1. Introduction

Infections with liver flukes have been occurring worldwide, and thus bear considerable veterinary and public health importance [1,2,3]. The giant liver fluke *Fascioloides magna* (Digenea: *Fasciolidae*) is originally a parasite of North American deer species, imported to Europe with infected white-tailed (*Odocoileus virginianus*) and wapiti deer (*Cervus elaphus canadensis*) [4,5]. Introducing non-native parasites to naïve hosts can lead to losses in livestock production. Furthermore, it negatively influences the survival of the local wildlife populations [6,7].

The life cycle of *F. magna* begins with the release of eggs by mature flukes and excretion in the feces of the mammalian host [5]. In water, the miracidia hatch from the eggs and actively search for the aquatic snail, the first intermediate host. In the snail, the parasite development passes through the sporocysts, rediae, and cercariae stages. The cercariae encyst as metacercariae on vegetation, thus becoming available for ingestion by the mammalian host. The ingested metacercariae exocyst and the juvenile flukes penetrate the intestinal walls. In this manner, they can reach the liver and migrate through the parenchyma. Metacercariae then encapsulate within pseudocysts and further develop into mature flukes [5].

Three types of final hosts are recognized: definitive, aberrant, and dead-end [4,5]. The usual definitive hosts for *F. magna* in Europe are red deer (*Cervus elaphus*), white-tailed deer (*Odocoileus virginianus*), and fallow deer (*Dama dama*) [5]. By shedding eggs through feces and possessing huge migration potential, definitive hosts are inevitably beneficial for the maintenance and spread of *F. magna* in nature. In dead-end hosts, the parasite usually fails to reach sexual maturity, as the detection of eggs in feces samples is not possible. In aberrant hosts, parasites cannot complete the life cycle in the liver, resulting in the detrimental dissemination of immature flukes, excessive organ damage, hemorrhages, and eventual death of the host [5].

Pathological changes, clinical signs, and outcomes of *F. magna* infection are strongly related to the type of final hosts and their different susceptibility. *Fascioloides magna* infection in North American natural definitive hosts is usually asymptomatic [8], although moderately poor body conditions have occurred in the red deer population [9]. The high prevalence of fascioloidosis in Croatia makes it significant for wildlife management [10,11,12]. In line with this fact, different approaches to improve the health status of the infected populations are under evaluation.

Advances in omics technologies, particularly proteomics, driven by the availability of new genomes sequencing, development of high-throughput mass spectrometry analysis, and boosts of bioinformatics tools for data analysis, are substantially contributing to studies on host–parasite interactions. These innovative approaches hold the opportunity to develop novel anti-trematode drugs and vaccine candidates in human and veterinary medicine [13,14,15,16].

The transcriptome and secreted proteome of the adult *F. magna*, collected from the naturally-infected livers of red deer, were characterized as laying the groundwork for future studies [17]. The complementary analysis of liver and serum proteome in wild boars, a dead-end host in *F. magna* infection, enabled insight into changes in the proteome profile of the host at the local and systemic level, revealing association with immune response, oxidative stress, and metabolomic changes in the liver of the host [18]. The excretory and secretory activity of the fluke has been the topic of several studies on animals infected with *Fasciola hepatica*, a parasite similar to *F. magna* [19,20,21,22]. A recent iTRAQ-based quantitative proteomic study explored serum changes in the definitive host, water buffaloes, infected with *Fasciola gigantica* [23]. There have been no previous proteomic host-related studies in red deer infected with *F. magna*.

Therefore, the current study aimed to analyze differences in liver proteomes between *F. magna*-infected and control, apparently healthy, red deer using a label-based high-throughput quantitative proteomics approach. In addition, the bioinformatic functional analysis of proteins with different abundances allowed for deciphering the mechanisms of host–parasite interactions.

## 2. Materials and Methods

### 2.1. Sample Collection

This study had an observational design. Liver samples from red deer were collected immediately following the regular hunting operations between March and August 2021. In total, the study involved 25 animals, including 12 animals (6 females and 6 males) which served as a control group, and 13 animals (8 males and 5 females) from the area where fascioloidosis is established (Baranja, Slavonija, and Moslavina). Within the disease control programme, triclabendazole-medicated baits were available to animals during spring. Age assessment relied on body development and tooth characteristics, and all animals were young adults older than 2 years [24]. Liver samples were collected following the evisceration of the animals. Parasitological examination of the liver is the gold standard for fascioloidosis diagnosis [25,26]. Each liver was sectioned as approximately 2-cm thick slices and thoroughly examined for traces of iron-porphyrin, fluke’s migratory channels, pseudocysts, and juvenile or adult flukes. Tissue samples were collected, stored in a plastic tube, signed, transported at 4 °C, and frozen at −20 °C (for no more than six months) until further analysis. A macroscopic examination of the other organs showed no abnormalities.

### 2.2. Sample Preparation

Proteomic analysis of liver samples was performed by a tandem mass tag (TMT)-based quantitative approach as described previously [18]. Cold-cut frozen liver samples (50 mg) were homogenized in 300 µL of lysis buffer (2% SDS in 0.1 M triethyl ammonium bicarbonate (TEAB, Thermo Scientific, Rockford, IL, USA)), followed by two cycles of sonication at maximum amplitude (Qsonica, Newtown, CT, USA) on ice. After centrifugation at 16,000× *g* for 20 min at 4 °C, the total protein concentration in the supernatant was determined using BCA assay (Thermo Scientific, Rockford, USA). Protein extracts were processed using the filter-aided sample preparation protocol with some modifications [27]. In brief, for each sample, an amount of 35 μg of total proteins was diluted to a volume of 200 μL with urea buffer (8 M urea in 0.1 M Tris–HCl pH 8.5), transferred to the 10-kDa membrane filter units (Microcon YM-10, Merck Millipore), centrifuged (13,000× *g*, 20 min, 20 °C), and subsequently washed with 100 μL of urea buffer. Proteins were alkylated (50 mM iodoacetamide, 20 min at room temperature in the dark), washed twice with urea buffer, twice with TEAB (100 mM pH 8.5), and digested by Trypsin gold (Promega, Madison, WI, USA, enzyme-to-protein ratio 1:35, *v*/*v*, at 37 °C overnight). In the next step, peptides were eluted from the filter by centrifugation and washed with 50 μL of TEAB/acetonitrile (ACN) (1:1, *v*/*v*). After that, the TMT 6plex reagents were prepared as described by the manufacturer (Thermo Scientific, Rockford, IL, USA). An amount of 19 μL of specific TMT label was added to each sample for labeling (60 min, room temperature) and quenched (15 min, room temperature) using 5% hydroxylamine (Sigma-Aldrich, St. Louis, MO, USA). In parallel, the same procedure was employed to prepare the internal standard using the pool of all samples. Five TMT-modified samples were randomly combined with the labeled internal standard, aliquoted, dried, and assessed via liquid chromatography with tandem mass spectrometry (LC-MS/MS) analysis. 

### 2.3. LC-MS/MS Analysis

Ultimate 3000 RSLCnano system (Dionex, Germering, Germany) coupled to a Q Exactive Plus mass spectrometer (Thermo Fisher Scientific, Bremen, Germany) was used for LC-MS/MS analysis. Following dissolving in loading solvent (2% ACN, 0.1% formic acid) and loading onto the trap column (C18 PepMap100, 5 μm, 100A, 300 μm × 5 mm), TMT-labeled peptides were desalted for 12 min at the flow rate of 15 μL/min. The gradient separation on the analytical column (PepMap™ RSLC C18, 50 cm × 75 μm) involved two mobile phases: A, consisting of 0.1% formic acid in the water, and B, which was 0.1% formic acid in 80% ACN. The percentage of phase B was linearly increased from 5% to 55% during the first 120 min. Further, the percentage was increased to 95% for 1 min, remained at 95% for 2 min, and re-equilibrated at 5% B for 20 min at the flow rate of 300 nL/min. The separated peptides were ionized using a Nanospray Flex ion source (Thermo Fisher Scientific, Bremen, Germany) with a 10 μm-inner diameter SilicaTip emitter (New Objective, Littleton, MA, USA). The MS was operated in positive ion mode using the DDA Top8 method. The *m/z* range for the full scan MS spectra acquisition was between 350 and 1800 with a resolution of 70,000, 120 ms injection time, AGC target 1 × 10^6^, a ± 2.0 Da isolation window, and the dynamic exclusion 30 s. HCD fragmentation was performed at step collision energy (29% and 35% NCE) with a resolution of 17,500 and AGC target of 2 × 10^5^. The fragmentation did not occur if the precursor ions were in the unassigned charge states or had a charge of +1 or more than +7. 

The identification and quantification of proteins followed the SEQUEST algorithm in Proteome Discoverer (version 2.3., ThermoFisher Scientific). The database was searched against *Cervidae* FASTA files (downloaded from Uniprot database on 9 July 2021, 105,671 sequences) according to the following criteria: two trypsin missed cleavage sites, precursor and fragment mass tolerances of 10 ppm and 0.02 Da, respectively; carbamidomethyl (C) fixed peptide modification, oxidation (M), and TMT sixplex (K, peptide N-terminus) dynamic modifications. The false discovery rate (FDR) for peptide identification was 1%. The presence of at least two unique peptides was necessary for protein identification. Protein quantification was accomplished by correlating the relative intensities of reporter ions extracted from tandem mass spectra to those of the peptides selected for MS/MS fragmentation. The internal standard allowed for the comparison of the relative quantification results between the sixplexes. Protein abundances were normalized to the total protein amount and scaled to the controls’ average to enable comparison inside one sixplex and between different sixplexes. The ProteomeXchange Consortium contains the mass spectrometry data available via the PRIDE partner repository with the dataset identifier PXD037544 [28].

### 2.4. Statistical and Bioinformatic Analysis

Statistical analysis was performed using R software v.4.1.2. [29], following the previously published in-house protocol [18]. In brief, after removing outliers and proteins with abundances missing in more than half of the samples, differences between groups were tested by non-parametric Mann–Whitney U test, with Benjamini-Hochberg false discovery rate (FDR) correction. Protein abundances were considered statistically significant for FDR < 0.05. Protein abundance fold changes between the two groups were calculated as median (*F. magna* infected group)/median (control group) and expressed on the log_2_ scale. The R package ggplot2 v3.1.1 [30] was used for the Principal Component Analysis (PCA) and volcano plots. 

For functional analysis, protein accession numbers were converted into Gene ID using the UniProt database conversion tool [31]. Using the UniProt BLAST tool, proteins with no gene ID available for *Cervus elaphus* and those annotated as uncharacterized were replaced with the *Bos taurus* orthologue when an identity higher than 70% was present. For functional GO classification, the Protein Analysis Through Evolutionary Relationship tool (PANTHER) (http://www.pantherdb.org/) with the subset of gene ontology (GO) terms (GO Slim database) was employed [32]. The Reactome tool enabled the pathway enrichment analysis, using the human genome as background and FDR-adjusted *p*-value < 0.05 for significantly enriched pathways [33].

## 3. Results

### 3.1. F. magna Infection Diagnosis and Liver Inspection

**Figure 1:**
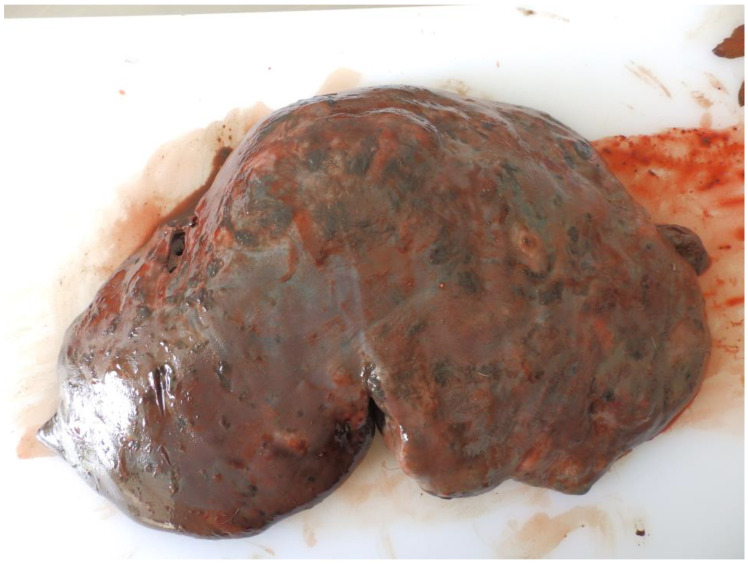
*F. magna*-positive red deer liver. Note: loss of translucency, traces of iron-porphyrin, and irregular surface due to underlying pseudocysts.

Livers of animals collected from endemic areas showed traces of iron-porphyrin (black pigment) on the surface and sections, fibrin deposits on Glisson’s capsule, loss of translucency, and irregular liver surface (Figure 1). In positive animals, numerous infection-specific features were present such as migratory channels, pseudocysts, and juvenile and adult flukes. Pseudocysts had well-developed walls with traces of calcification on the surface. Nevertheless, if cyst degradation took part, parasites were indistinguishable from the homogenous mass inside the cyst (Table 1). By contrast, the livers of uninfected control animals appeared normal, without any pathological changes, and were free of *F. magna* flukes.

### 3.2. Proteomics Analysis

**Figure 2:**
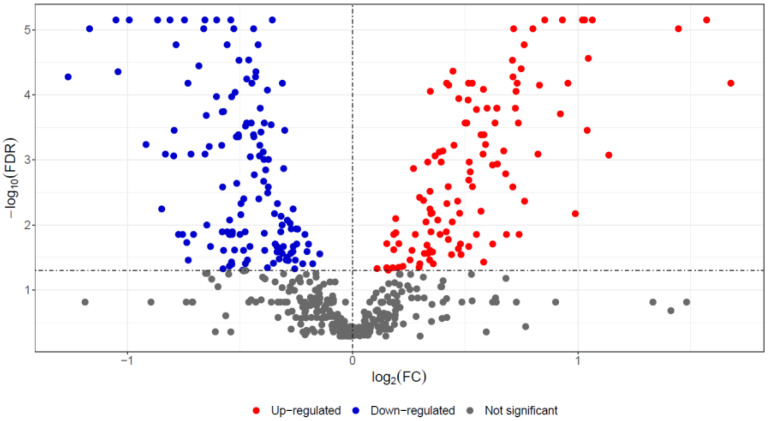
Volcano plot showing proteins with differential abundance between control and *F. magna*-infected groups after FDR *P*-value correction.

**Figure 3:**
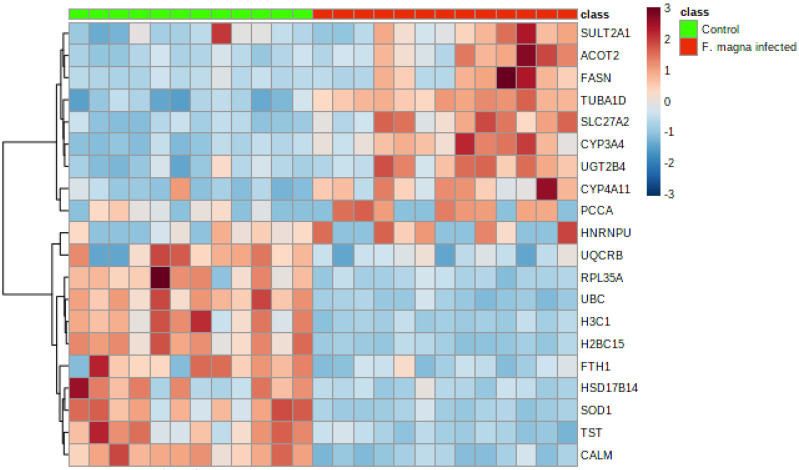
Hierarchical cluster analysis (HCA) based on the top 10 proteins with the highest and lowest significantly differential abundances between control (green panel) and *F. magna*-infected (red panel) red deer (*Cervus elaphus*) using Euclidean as a distance measure and ward as a clustering algorithm. Each colored cell on the map corresponds to the abundance, with the red color meaning an increased, and blue a decreased abundance. Full protein names for gene IDs can be found in the Table 2.

In liver samples of red deer, 550 master proteins were identified and quantified by a label-based quantitative proteomic approach, according to set criteria (two unique peptides and 1% FDR). After the exclusion of proteins with more than 50% missing values, significant differences in abundances between the *F. magna*-infected and control groups were assessed by the Mann–Whitney test (FDR < 0.05) and were found for 234 proteins (Table 2, Supplementary Table S1). Of those, 123 had higher abundance, and 111 had lower abundance in the *F. magna*-infected group compared to the control group, as depicted in the below volcano plot (Figure 2). Selected proteins with the highest fold change differences are also presented as heat map (Figure 3).

**Figure 4:**
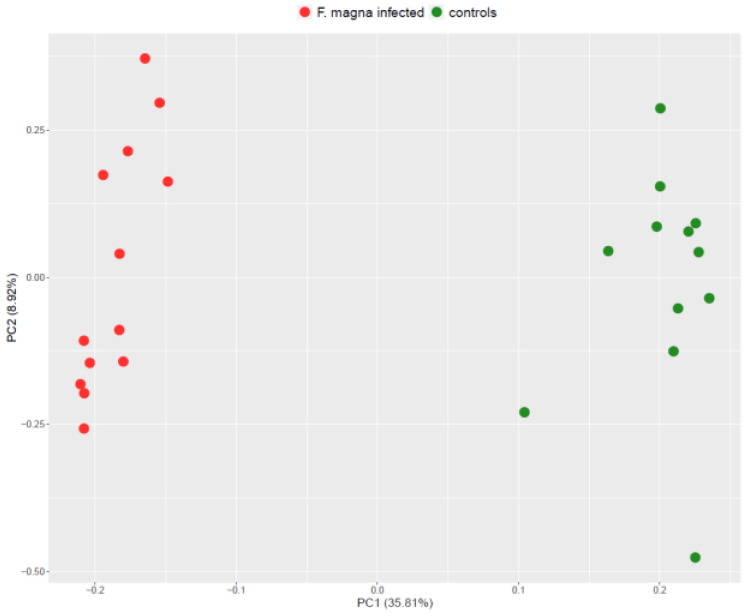
Principal component analysis (PCA) score plot showing the distribution of samples from control (green dots) and *F. magna*-infected (red dots) groups.

PCA score plots revealed a clear separation between the control and infected groups (Figure 4). The clusters were separated based on the principal component 1 (PC1), which captured 35.8% of the variance in the dataset, while PC2 captured 8.92% of the variance.

### 3.3. Functional Enrichment Analysis

**Figure 5:**
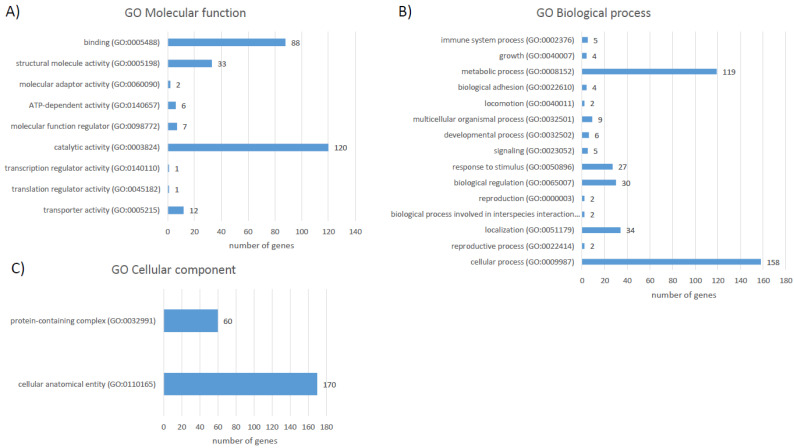
Gene Ontology analysis for the proteins with different abundance between the control and *F. magna*-infected groups using the PANTHER GO-Slim analysis: (**A**) molecular function; (**B**) Biological Process; (**C**) Cellular Component.

Protein accession IDs were matched to 231 unique gene IDs for the functional analysis of differentially abundant proteins. According to PANTHER GO Slim analysis, liver proteins with significant differences in abundances within the control and *F. magna*-infected groups were involved in cellular processes (158 genes, 56.4%), metabolic processes (119 genes, 42.5%), biological regulation (30 genes, 10.7%), response to stimulus (27 genes, 9.60%), and others (Figure 5B). 

Cellular localisations of proteins were mainly annotated as cellular anatomical entities (170 genes, 60.7%) with domination of the intracellular anatomical structure, cytoplasm, organelle, and membrane proteins (Figure 5C). The molecular functions of proteins with significantly differential abundances were catalytic activity (120 genes, 42.9%), binding (88 genes, 31.4%), structural molecule activity (33 genes, 11.8%), and others (Figure 4A).

**Figure 6:**
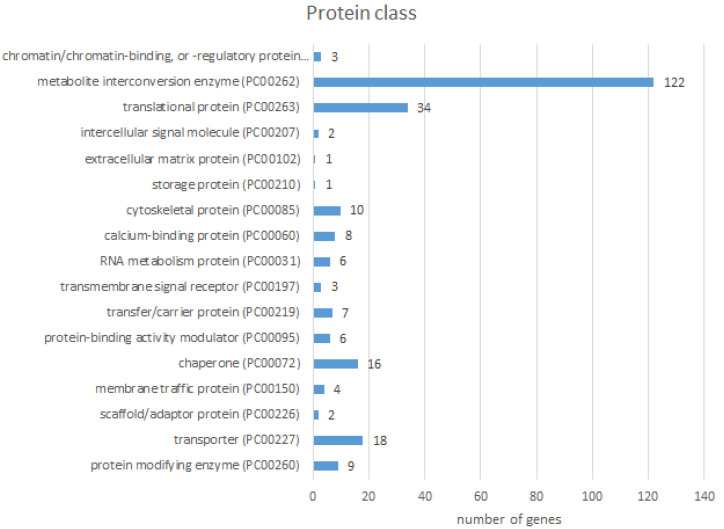
Protein Class analysis of proteins with different abundances between the control and *F. magna*-infected groups performed by PANTHER GO-Slim analysis tool.

The largest part of proteins with differential abundance belonged to metabolite interconversion enzymes (122), following by translational proteins (34), transporters (18), chaperons (16), and cytoskeletal proteins (10) (Figure 6). 

**Figure 7:**
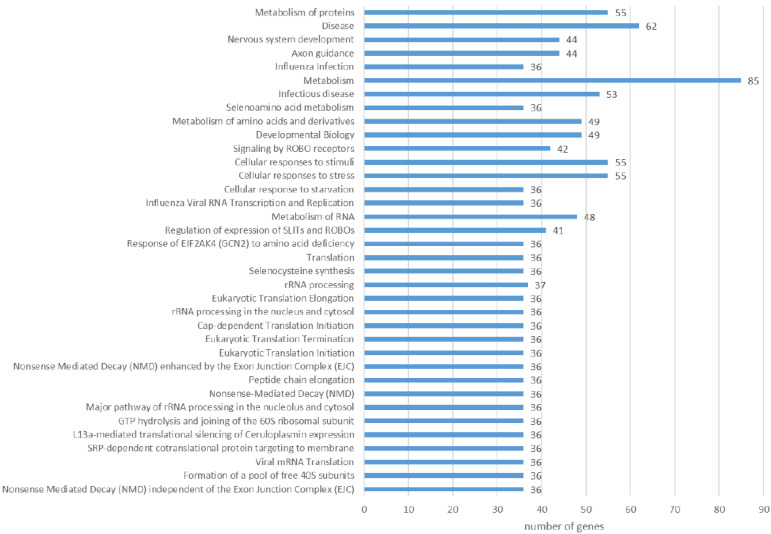
Reactome pathways enriched from liver proteins with lower abundance in *F. magna*-infected group compared to control (FDR < 0.05, minimum of 35 genes per pathway).

**Figure 8:**
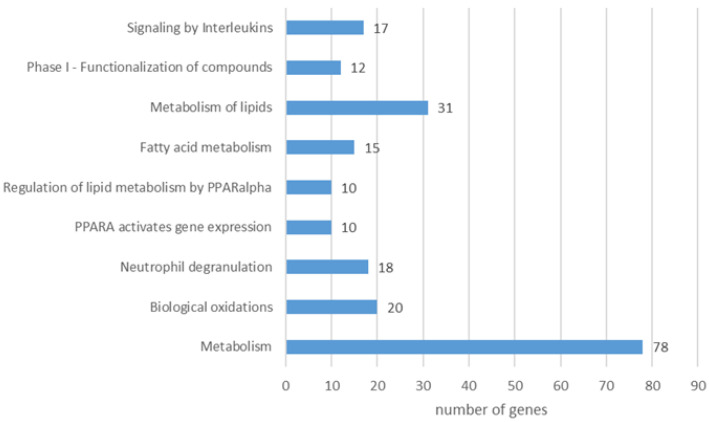
Reactome pathways enriched from liver proteins with higher abundance in *F. magna*-infected group compared to control (FDR < 0.05, minimum of 10 genes per pathway).

The Reactome pathway analysis of the liver proteins differing in abundance revealed 227 pathways (FDR < 0.05). After reducing the total number by including only those with a minimum of 35 proteins, 37 remained (Supplementary Table S2). Among the most representative were those associated with metabolism (metabolism in general, metabolism of proteins, and metabolism of amino acids and derivatives), immune system (cellular responses to stress, cellular responses to stimuli, infectious disease, and innate immune system), translation, and related pathways (metabolism of RNA, translation, rRNA processing, and eukaryotic chain elongation), signaling (signaling by ROBO receptors) and others. When analyzing only proteins with a lower abundance in the infected group compared to the control, the enrichment was present for 36 pathways (FDR < 0.05, minimum 35 genes per pathway), with the most distinguished ones being: metabolism (85 genes), metabolism of proteins (55 genes), cellular response to stress (55 genes), infectious disease (53 genes), metabolism of RNA (48 genes), and translation (36 genes) (Figure 7). A closer look at proteins with higher abundance in the infected group revealed nine pathways (FDR < 0.05, minimum 10 genes per pathway): metabolism (78 genes), metabolism of lipids (31 genes), biological oxidations (20 genes), neutrophil degranulation (18 genes), signaling by interleukins (17 genes), fatty acid metabolism (15 genes), phase I - Functionalization of compounds (12 genes), PPARA activates gene expression (10 genes), and regulation of lipid metabolism by PPAR alpha (10 genes) (Figure 8).

## 4. Discussion

Deciphering the host–parasite interactions in wild animals is a prerequisite for predicting how the expansion of existing and the emergence of new parasites affects population management and dynamics. Considering the tropism of *F. magna*, the alterations in biological pathways within hepatic tissue should be the primary target for investigation. The present study contributed the first data about the liver proteome changes in red deer infected with *F. magna*, obtained via the comprehensive high-throughput TMT-based proteomics approach. Altogether, almost half of 550 identified proteins had altered abundance in the *F. magna*-infected group compared to the control group, with higher abundance evidenced for 123 proteins, and lower for 111 proteins. These proteome changes were linked with metabolism, the immune system, translation, and signal transduction.

Red deer serve as the definitive host for *F. magna* infection. The liver offers a favorable immunological environment for parasites who have developed complex and multifaceted mechanisms which modulate the host response in order to counter infection and repair tissue damage [34,35]. Our results allowed for in-depth molecular profiling of (patho)biological pathways accompanying *F. magna* infection in the definitive host. The iron-porphyrin presence is characteristic only for *F. magna* infection in Europe. Therefore, visual inspection with a thorough analysis of a 2 cm-thick liver slice is highly accurate for detailing the diagnostics of *F. magna* infestation [25,26].

A study on the liver proteome of wild boars infected with *F. magna* showed that all differential proteins were less abundant in the infected boars when compared with those without infection. Proteome changes in this dead-end host for *F. magna* were associated with metabolism and cellular energy production [18]. According to the current study, the changes in the liver proteome of *F. magna* definitive host were more extensive, affecting more than 50% of the proteome, with the changes being in both directions in comparison to the uninfected deer. Proteins with lower abundance in the infected group were mainly associated with RNA metabolism, translation and transcription processes, and protein metabolism. The predominant pathways among significantly different proteins with higher abundance were associated with fatty acid metabolism, biological oxidations, and signaling cascades. Nevertheless, caution is necessary for the interpretation, as we studied naturally-infected free-ranging deer with different stages of infection and variability between individual animals, thereby representing potential limitations of the present study.

### 4.1. Alterations in Translation Processes during F. magna Infection

Most of the differentially abundant proteins with lower abundance in the *F. magna*-infected group belonged to ribosomal proteins (19 proteins of large S60 ribosomal subunit and 12 proteins of small 40S ribosomal subunit), nucleosome components (histone H2B (H2BC15), histone H1.0 (H1-0)), ribonucleoproteins (heterogeneous nuclear ribonucleoprotein H isoform X5 (HNRNPH1), RRM domain-containing protein (HNRNPL)), proteasome subunits (proteasome subunit beta (PSMB4, PSMB5, and PSMB6)), and polyubiquitin (UBC). Apart from protein synthesis, many ribosomal proteins regulate apoptosis and the cell cycle [36]; therefore, their role during parasitic infection merits further assessment. The translational activity is very intensive in the immature stages of *F. hepatica* [19]. In that context, the most enriched GO terms related to transcription and translation could mirror the intensive growth and development of flukes in the liver. 

The selective degradation of proteins, occurring through the ubiquitin-proteasome system (UPS), is critical for most cellular processes, such as the cell cycle, cellular response to stress and extracellular modulators, modulation of cell surface receptors, ion channels, secretory pathway, and DNA repair [37,38]. The UPS plays a key role in linking the cell cycle with metabolic activities [39]. The alteration of polyubiquitin and abundance of proteasome subunits in fascioloidosis suggested that protein turnover control serves as a mechanism of the host response to environmental changes caused by *F. magna*.

### 4.2. Alterations in Liver Metabolism during F. magna Infection

In this study, a lower abundance of various enzymes included in carbon metabolism, tricarboxylic acid (TCA) cycle, and oxidative phosphorylation (fumarate hydratase (FH), transaldolase (TALDO1), glyceraldehyde-3-phosphate dehydrogenase (GAPDH), triosephosphate isomerase (TPI1), phosphoglycerate mutase (PGAM1), aspartate aminotransferase (GOT1, GOT2), ATP synthase subunit alpha (ATP5F1A), ATP synthase subunit beta (ATP5F1B), 2-phospho-d-glycerate hydro-lyase (ENO1), cytochrome c oxidase subunit (COX6B1), and cytochrome b5 (CYB5A)) was demonstrated in the *F. magna*-infected group. These results are concordant with a previous report which showed altered energy production in the liver of *F. magna*-infected wild boars [18]. Also, in buffaloes infected with *F. gigantica*, the downregulation of metabolism-related processes in the liver was prominent across all time points [23].

We also found a higher abundance of TCA enzymes (glutamate dehydrogenase, (GLUD), malate dehydrogenase, (MDH1), D-3-phosphoglycerate dehydrogenase (PHGDH), and succinate--CoA ligase (SUCLG2)), components of the oxidative phosphorylation pathway (cytochrome c oxidase polypeptide Va (COX5A), sodium/potassium-transporting ATPase subunit alpha (ATP1A1), flavoprotein-ubiquinone oxidoreductase (ETFDH), succinate dehydrogenase (SDHA, SDHB), and cytochrome b-c1 complex subunit 1 (UQCRC1)) and enzymes included in glycogen metabolism (4-alpha-glucanotransferase (AGL), alpha-1,4 glucan phosphorylase (PYGL), and pyruvate carboxylase (PC)). A substantial number of proteins with higher abundance in the *F. magna*-infected group were related to the metabolism of lipids and fatty acid metabolism, including carrier proteins (SLC25A1, SLC25A20, SLC25A4, SLC27A2, SLC2A2, and SLCO1B3), fatty acid synthetase (FASN), enzymes included in beta-oxidation (very long-chain specific acyl-CoA dehydrogenase (ACADVL), carnitine O-palmitoyltransferase (CPT1A), long-chain specific acyl-CoA dehydrogenase (ACADL), acyl-coenzyme A oxidase (ACOX1), enoyl-CoA hydratase and 3-hydroxy acyl CoA dehydrogenase (EHHADH), propionyl-CoA carboxylase beta chain (PCCB), acyl-coenzyme A thioesterase 1-like (ACOT2), and propanoyl-CoA:carbon dioxide ligase subunit alpha (PCCA)), enzymes of the fatty acid biosynthesis pathway (acyl-CoA synthetase medium chain family member 3 (ACSM3), and medium-chain acyl-CoA ligase (ACSF2)), and other related proteins (glutathione transferase (GSTM4), cytochrome P450 members (CYP4A11, CYP3A4), terpene cyclase/mutase (LSS), 3-hydroxy-3-methylglutaryl coenzyme A synthase (HMGCS2), monoglyceride lipase (MGLL), and sulfotransferase (SULT2A1)).

During their development in the liver, immature flukes transitioned from relying on endogenous energy sources to a dependence on the host for nutrients. They have a highly-reduced lipid metabolism due to the inability to process beta-oxidation [40]. Mitochondrial fatty acid beta-oxidation is a catabolic process which breaks down fatty acids into acetyl-CoA and allows for the production of energy. The abundance of proteins associated with beta-oxidation and lipid metabolic processes underlines metabolic perturbations in the liver of infected red deer due to parasite presence. Long-chain fatty acids are the essential energy source for liver flukes [41]. Furthermore, the most well-known transcriptional regulators of beta-oxidation are the peroxisome proliferator-activated receptors (PPARs) [42], and 2 PPAR-related pathways (PPARA activates gene expression and regulation of lipid metabolism by PPAR alpha) were found to be significantly enriched in this study.

### 4.3. Host-Parasite Interface during F. magna Infection

Cathepsin D (CTSD), an aspartic endopeptidase, and calmodulin (CALM), a calcium-binding protein with versatile roles, were among the identified proteins known to participate in the host-parasite interactions. Cathepsin peptidases, active against both host and parasite proteins, represent a prominent feature of the *F. hepatica* secretome [19,21,22,43]. Through degradation of the extracellular matrix (ECM), the cathepsins facilitate parasite migration in the liver and contribute to pathogenicity. Via binding the calcium ions, CALM interplays in numerous biological processes such as cytoskeleton architecture and function, the activation of phosphorylase kinases, protein folding, ion transport, and stress responses [44]. Lower abundances of these proteins in our study might have originated from an increased consumption due to the degradation of the extracellular matrix and host response to *F. magna* infection. 

Annexins are the ubiquitous phospholipid- and membrane-binding proteins necessary for the cell membrane transport and repair, cell adhesion, signal transduction [45], the modulation of inflammation, and fibrinolytic homeostasis [46]. Their involvement in host–parasite interactions marked parasite annexins as potential therapeutic targets [47]. Annexins provide binding platforms on membranes for structural stabilization or enzymatic activities. Several annexins were found in this study (ANXA1, ANXA2, ANXA4, and ANXA5) with predominantly higher abundance in the infected group, indicating the importance of this versatile class of proteins in parasite–host interaction. 

Heat shock proteins (HSPs) are a large family of immunogenic molecular chaperones. Their evolutional conservation implies their substantial involvement in the immune response against pathogens [48,49]. They link innate and acquired immune responses by enhancing the release of cytokines and chemokines, accompanied by dendritic cells and lymphocyte activation [50,51]. Several members of HSP families were found with increased abundance in red deer infected with *F. magna* (HSP90AA1, HSP90AB1, HSP90B1, HSPA9, HSPD1) compared to controls, indicating host immune response and tissue damage due to *F. magna* infection.

### 4.4. Fibrosis-Related Protein Alterations during F. magna Infection

Owing to the capability of *F. magna* to evade immune mechanisms, fascioloidosis persists as a chronic infection. Parallel with the continuous inflammatory injury, the ECM accumulates in the hepatic parenchyma, thus progressively replacing functional liver tissue and resulting in fibrosis [52]. The study results include several proteins associated with fibrosis, cytoskeleton remodeling, and ECM deposition, thus adding several details about liver fibrosis to the knowledge of *F. magna* infection associated with red deer. 

Plastin 2 (LCP1) is an actin-binding protein that is involved in the plasticity of the actin cytoskeleton in response to external signals or during cell migration and adhesion [53,54]. Furthermore, it shows immunomodulatory features such as involvement in T cell activation [55]. Tubulins (TUBA1D and TUUB5), the major constituents of the cytoskeleton, are also indispensable in cytoskeleton (re)organization. Alpha actinin 4 (ACTN4) modulates the cytoskeleton and cell motility by cross-linking actin molecules [56]. Nevertheless, it is also an immunity-involved protein, which increases the transcriptional activity of nuclear factor kappa B (NF-κB) in the presence of tumor necrosis factor-alpha (TNF-α) [57]. Spectrin beta chain (SPTBN1) associates the molecular function with ACTN [58]. Through actin cross-linking and serving as a molecular scaffold protein, it connects the plasma membrane to the actin cytoskeleton and assists in cell shape establishment, arrangement of transmembrane proteins, cell adhesion, and spreading [59]. It interacts with calmodulin, thus being a candidate for the calcium-dependent cytoskeleton movement on the membrane [60]. Myosin-9 (MYH9), myosin light polypeptide 6 (MYL6), and tropomyosin alpha-4 chain isoform (TPM4) are also members of cytoskeleton reorganization protein machinery, with a responsibility of stabilizing cytoskeleton actin filaments.

Filamin B (FLNB) is another fibrosis-related protein found in our study. It connects cell membrane glycoproteins to the actin cytoskeleton [52]. A lower abundance of collagen alpha-1(VI) chain (COL6A1), another ECM component, was found in the infected group. The Rho GTPase family is accountable for the regulation of cytoskeletal dynamics and cell movement via specific signal transduction pathways [61,62]. Two members of this family, ras GTPase-activating-like protein (IQGAP2) and rho GDP-dissociation inhibitor 1 (ARHGDIA), were identified in this study, with roles in cell motility regulation and interaction with calmodulin. 

Liver fibrosis represents an in situ response to flukes’ migration through the liver and the continuous remodeling process, characterized by the excessive deposition of ECM in the hepatic parenchyma. The abundance alteration of all these fibrosis-related proteins suggested remodeling of the actin cytoskeleton in response to *F. magna* infection.

### 4.5. Oxidative Stress and Drug Metabolism Changes during F. magna Infection

Oxidative stress is a hallmark of fascioloidosis, caused by the host immune response, parasite migration, and induction of fibrogenesis [63]. Reactive oxygen species act as the first line of the innate immune response; nevertheless, when present in excessive amounts, their functions become detrimental and contribute to the progression of the disease [64]. Previous studies reported that infection with *F. hepatica* reduces the antioxidant potential in humans [65] and rats [66,67]. In the present study, sulfotransferase (SULT2A1), annexins (ANXA1, ANXA2, and ANXA5), glutathione transferase (GSTM4), thioredoxin domain-containing protein (PRDX2), and ceruloplasmin (CP) had higher abundance in the infected group than in control animals. The results were the opposite for superoxide dismutase (SOD1), sulfurtransferase (TST), glutathione S-transferase (GSTM3), thioredoxin-dependent peroxide reductase (PRDX3), and peroxiredoxin-like 2 (PRXL2A). We reported SOD1 among the proteins with the most protruding fold change difference in proteins with lower abundance. Our result corroborates with lower SOD activity measured in the liver and serum in *F. magna*-, *F. hepatica*-, and *F. gigantica*-infected hosts [18,19,66]. Also, a high number of proteins included in the biological oxidations pathway evidenced that *F. magna* infection threatened to multiply the oxidative stress in the study animals.

Furthermore, several members of the cytochrome P450 family (CYB5R3, CYP3A4, CYP4A11, and CYB5A) showed altered abundances, alongside other proteins which are responsible for xenobiotics detoxication (catechol O-methyltransferase, NADPH-cytochrome P450 reductase, MOSC domain-containing protein, dimethylaniline monooxygenases, S-adenosylmethionine synthase, and UDP-glucuronosyltransferase). The control of liver fluke infections has relied predominantly on treatment with anthelmintic drugs in endemic areas. Unfortunately, the potent flukicidal agents’ intensive usage has resulted in resistance development. Numerous in vitro and in vivo experiments highlighted the role of the CYP450 system in resistance/susceptibility to antiparasitic drugs [68,69,70]. Previous studies reported a lower abundance of various CYP isoforms in the livers of *F. magna*-infected wild boars and *F. hepatica*-infected sheep [18,71]. Also, the xenobiotics biotransformation enzymes (flavin-containing monooxygenases (FMO), CYP450 monooxygenases, cyclooxygenases, and monoamine oxidases) participated in the pathways highlighted by our results. The FMO enzyme system appears to be the principal metabolic pathway involved in the drug metabolism in both triclabendazole-susceptible and -resistant flukes [72]. Our results support the idea of these proteins’ involvement in drug resistance development, which might be an attractive hypothesis for further studies.

## 5. Conclusions

A quantitative high-throughput proteomics approach identified a complex network of numerous alterations in the liver proteome of red deer (*Cervus elaphus*) infected by the giant liver fluke *Fascioloides magna*. The pathophysiological mechanisms of these changes existed at different levels—from metabolic and immunological perturbations, allowing the successful survival of the parasite in its definitive host, to detrimental changes in the liver tissue architecture. Apart from extending the knowledge about the studied host–parasite interactions, the identified network holds the potential to target the biological pathways which are potentially responsible for the adverse effects of the pharmacological agents currently used for the treatment and control of liver fluke infections.

## Figures and Tables

**Table 1:** Results of macroscopic and parasitological examination of liver samples from infected red deer.

	Migration	Juvenile Fluke	Adult Fluke	Pseudocyst	Destroyed Pseudocyst
Sample 1	0	0	2	1	0
Sample 2	1	0	2	1	1
Sample 3	1	1	2	1	0
Sample 4	3	1	8	8	7
Sample 5	2	12	1	3	65
Sample 6	0	5	12	5	0
Sample 7	3	21	25	6	0
Sample 8	1	1	0	0	78
Sample 9	6	6	4	11	11
Sample 10	5	23	59	8	0
Sample 11	0	1	2	1	1
Sample 12	1	5	44	22	5
Sample 13	7	3	0	14	7

**Table 2:** Top 10 proteins with the highest and lowest significantly differential abundances between control and *F. magna*-infected red deer (*Cervus elaphus*) identified and quantified using tandem mass tags (TMT) proteomics approach in the liver.

Accession	Gene ID	Description	*p* Value	FDR	FC	log2FC
Proteins with higher abundance in *F. magna* group						
A0A6J0WZS6	SLC27A2	Very long-chain acyl-CoA synthetase-like	<0.001	<0.001	2.77	1.47
A0A6J0W5Y4	CYP3A4	Cytochrome P450 3A24	<0.001	<0.001	2.08	1.06
A0A6J0ZFP6	CYP4A11	Taurochenodeoxycholic 6 alpha-hydroxylase-like isoform X2	<0.001	<0.001	2.08	1.06
A0A5N3V0H2	UGT2B4	UDP-glucuronosyltransferase	<0.001	0.001	1.94	0.96
A0A6J0YYJ9	ACOT2	Acyl-coenzyme A thioesterase 1-like isoform X1	<0.001	0.001	1.84	0.88
A0A6J0XYD9	PCCA	Propanoyl-CoA:carbon dioxide ligase subunit alpha	<0.001	0.001	1.84	0.88
A0A5N3XTI4	HNRNPU	Heterogeneous nuclear ribonucleoprotein U (Scaffold attachment factor A) **	0.009	0.014	1.77	0.82
A0A5J5MHR2	TUBA1D	Tubulin alpha chain	<0.001	<0.001	1.73	0.79
A0A212DC96	SULT2A1	Sulfotransferase (Fragment)	0.013	0.019	1.70	0.76
A0A212DAL6	FASN	3-hydroxyacyl-[acyl-carrier-protein] dehydratase	0.029	0.035	1.67	0.74
Proteins with lower abundance in *F. magna* group						
A0A6J0WX68	H3C1	Histone H3.1 **	<0.001	<0.001	0.50	−0.99
A0A5N3V8G4	UQCRB	Cytochrome b-c1 complex subunit 7	<0.001	<0.001	0.50	−1.00
A0A6J0XG22	UBC	Polyubiquitin-C	<0.001	<0.001	0.48	−1.05
A0A6J0Z5P0	RPL35A	60S ribosomal protein L35a	<0.001	<0.001	0.48	−1.05
A0A6J0WHA7	TST	Sulfurtransferase	<0.001	<0.001	0.47	−1.10
A0A6J0X0S3	HSD17B14	17-beta-hydroxysteroid dehydrogenase 14	0.004	0.007	0.46	−1.13
A0A5N3WJW3	CALM	Calmodulin **	<0.001	<0.001	0.46	−1.13
A0A5N3W733	FTH1	Ferritin	<0.001	<0.001	0.35	−1.51
O46412	SOD1	Superoxide dismutase [Cu-Zn]	<0.001	<0.001	0.35	−1.52
A0A6J0YAN6	H2BC15	Histone H2B	<0.001	<0.001	0.29	−1.77

** after blasting in UniProt.

## Data Availability

The mass spectrometry proteomics data have been deposited to the ProteomeXchange Consortium via the PRIDE partner repository with the dataset identifier PXD037544.

## Supplementary Materials

The following supporting information can be downloaded at: https://www.mdpi.com/article/10.3390/pathogens11121503/s1, Table S1: Proteins with differential abundances between control and *F. magna* infected red deer (*Cervus elaphus*) identified and quantified using tandem mass tags (TMT) proteomics approach in the liver; Table S2: Reactome pathways enriched from liver proteins significantly differential in abundance between control and *F. magna* infected red deer (FDR < 0.05).
